# Depression negatively affects patient-reported knee functional outcome after intraarticular hyaluronic acid injection among geriatric patients with knee osteoarthritis

**DOI:** 10.1186/s13018-019-1419-z

**Published:** 2019-11-27

**Authors:** Yu-Pin Chen, Yu-Yun Huang, Yueh Wu, Yi-Jie Kuo, Chung-Ying Lin

**Affiliations:** 1 Department of Orthopedic Surgery, Wan Fang Hospital, School of Medicine, College of Medicine, Taipei Medical University, Taipei, Taiwan; 2 Graduate Institute of Nursing- Linkou campus, Chang Gung University of Science and Technology, Taoyuan, Taiwan; 3 Department of Rehabilitation Sciences, Faculty of Health and Social Sciences, The Hong Kong Polytechnic University, 11 Yuk Choi Rd, Hung Hom, Hong Kong

**Keywords:** Hyaluronic acid, Anxiety, Depression, Knee osteoarthritis, Pain

## Abstract

**Purpose:**

Intraarticular hyaluronic acid injection (IAHA) is a popular treatment for knee osteoarthritis (OA). This study investigates whether depression, anxiety, and pain affect self-reported knee function in geriatric OA people who have received IAHA.

**Methods:**

Through convenience sampling, 102 geriatric patients (mean age = 70.91 ± 7.19; 28 males) with knee OA who had undergone IAHA participated in this study. All participants self-reported depression using the Geriatric Depression Scale (GDS), anxiety using the State-Trait Anxiety Inventory (STAI), knee function using the Western Ontario and McMaster University Osteoarthritis Index (WOMAC) and the International Knee Documentation Committee subjective knee evaluation form (IKDC), and pain severity using the Visual Analogue Scale (VAS). They completed the aforementioned questionnaires at baseline before injection and then again at 2-, 4-, and 6-month follow-ups.

**Results:**

Depression was significantly associated with IKDC, WOMAC physical function subscale, and total WOMAC scores. Anxiety was only significantly associated with the WOMAC pain subscale score. Pain severity was significantly associated with IKDC, WOMAC stiffness subscale, WOMAC physical function subscale, and total WOMAC scores.

**Conclusion:**

Given that depression negatively affected patient-reported knee function among geriatric OA patients who had undergone IAHA, further attention should be paid to the depressive status of this population.

## Background

Osteoarthritis (OA) commonly affects middle-age to elderly people, with an incidence of symptomatic knee OA up to 10% of men and 13% of women aged 60 years or older [1–3]. Although the end stages of progressive knee OA are generally managed by joint replacement, there are still various conservative treatments before end stage of knee OA with diverse short-term effects [4]. Thus, healthcare providers should understand and evaluate all the possible advantages and disadvantages among different treatments to make the optimal decision-making. One common conservative treatment, intraarticular hyaluronic acid injection (IAHA), improves joint lubrication, increases synovial fluid viscosity, and reduces pain by its analgesic and anti-inflammatory effects [5, 6]. However, controversy exists on the efficacy of IAHA especially in the geriatric population, who are vulnerable to chronic pain as a result of knee OA [7]. In fact, younger patients with less severe structural damage are reported to gain the best benefit from IAHA treatment than the elderly population [8]. To maximize the treatment effects of IAHA, factors that potentially hinder those treatment effects for the elderly must be investigated. Specifically, we proposed to investigate the roles of psychological health among these patients as the effects of psychological health on knee functions are understudied in the patients with knee OA.

Moreover, knee OA is reportedly associated with depressive symptoms especially among geriatric population [9]. Approximately 10% of the geriatric population suffers from severe anxiety and depressive symptoms [10]. Indeed, a study in six European countries has demonstrated that severe and stable joint pain is associated with anxiety and depressive symptoms among older adults with knee OA [11]. Moreover, depression is known to be a significant contributor to poorer health outcomes and to be the leading cause of disease burden worldwide [12]. According to the literature, functional outcomes in orthopedic procedure are also affected by depression, including lumbar spine surgeries [13], hip and knee arthroplasty [14, 15], shoulder surgery [16], and upper extremity pathologies [17, 18]. However, to the best of our knowledge, there is a lack of reports on psychological factors associated with the self-reported outcomes in geriatric OA people undergoing IAHA

Given that the self-reported psychological symptoms of an individual affect his/her quality of life [19], we hypothesized that such symptoms (depression and anxiety in this study) have the same effects on knee functions, as reported by geriatric people who have undergone IAHA. In addition to psychological symptoms, pain is another important concern of people with OA [20]. Pain catastrophizing, broadly defined as a negative orientation toward actual or anticipated painful experiences [21], is reported to be significantly correlated with health-related quality of life in patients with knee OA [22]. Moreover, deterioration of pain for geriatric OA patients is also associated with the future loss in walking ability [23]. Therefore, we additionally hypothesized that pain also influences the knee functioning that is reported by geriatric people who have undergone IAHA.

This study used a longitudinal design to determine whether depression, anxiety, and pain that affect the patient-reported knee functions of OA patients who were older than 60 years and had undergone IAHA.

## Material and methods

### Study design

Patients who had been diagnosed with knee OA and attended orthopedic clinics for IAHA in one hospital in Taipei, Taiwan, were prospectively recruited from June 2016 to April 2018. Inclusion criteria of the eligible participants were (1) aged 60 years or above, (2) had suffered symptoms of knee OA, including pain and stiffness, for at least 3 months, and (3) had radiographic OA grades 2–3 on the Kellgren and Lawrence grading scale [24]. Exclusion criteria were (1) had past or present trauma of, or surgery for cancer, malignant tumor or an infection in the target knee, (2) had a history of vasovagal shock and had used nonsteroidal anti-inflammatory drugs (NSAIDs) within 2 days prior to HA injection, (3) had received corticosteroids by injection in the target knee in the preceding 6 months, or (4) had cognitive impairment, identified by the Short Portable Mental Status Questionnaire (SPMSQ) with a cut-off of five errors [25].

After written informed consent had been obtained, the basic demographic data (age, gender, marital status, educational level, and underlying comorbidities) of each patient were collected using a face-to-face interview. All participants completed the questionnaires mentioned below at baseline (before injection) and at three follow-ups (at 2, 4, and 6 months). The questionnaires used the Geriatric Depression Scale (GDS), the State-Trait Anxiety Inventory (STAI), the Western Ontario and McMaster University Osteoarthritis Index (WOMAC), the International Knee Documentation Committee subjective knee evaluation form (IKDC), and the Visual Analogue Scale (VAS) of pain severity. Once they had completed the baseline survey, all participants underwent IAHA with a low-molecular-weight product of 2 mL sodium hyaluronate (SciVision Biotech Inc., Taiwan) with a molecular weight of 500–730 kDa once per week for 3 weeks. The protocol of IAHA is based on the manufacturing recommendations (http://www.scivision.com.tw/en/products_01_show.php?pc=4&ps=1), which is a general treatment protocol in Wan Fang Hospital. At the follow-ups, all participants were permitted to choose simultaneous treatment with COX-II inhibitors (etoricoxib 60 mg once daily) or physical therapy. Other treatments, including intraarticular injection with a steroid or platelet-rich plasma, were not permitted.

The entire protocol and instrumentation were approved by the ethical committee at Taipei Medical University, and the approval was registered as TMU-JIRB N201606003. All participants consented to the study and publication of data.

## Instruments

### Measurement of psychological symptoms

The GDS with 15 items is a validated tool to assess depression in older people by self-reporting [26, 27]. The state-anxiety subscale of the STAI with 20 items is a validated tool to assess subjective and transitory feelings of tension and nervousness by self-reporting [28]. A higher score on the GDS or the STAI indicates greater depression or anxiety, respectively.

### Measurement of knee function

The self-reported WOMAC with 24 items has three subscales (pain, stiffness, and physical function) and has promising psychometric advantages [29]. The self-reported IKDC with 18 items involves questions concerning symptoms, sports activities, and knee function. The IKDC also has promising psychometric benefits [30, 31]. A higher WOMAC or IKDC score indicates worse or better knee function, respectively.

### Measurement of pain

The VAS is an instrument that is regularly used to measure pain intensity [32]. If both knees are injected, then the VAS score for the left side is recorded first and then that for the right side is recorded.

## Statistical analysis

In addition to descriptive statistics for participant characteristics, numerous linear mixed effects models, which use restricted maximum likelihood estimation, were applied to understand the associations between patient-reported outcomes (pain, anxiety, and depression) and knee functions (measured using IKDC and WOMAC). All of these models control for the time effects of the hyaluronic acid, gender, marital status, comorbidities, and age. Moreover, independent *t* tests were applied to examine the outcome differences between patients who were grade 2 on the Kellgren and Lawrence grading scale and those who were grade 3. IBM SPSS 23.0 (IBM corp. Armonk, NY, USA) was used to conduct all of the analyses.

## Results

**Fig. 1:**
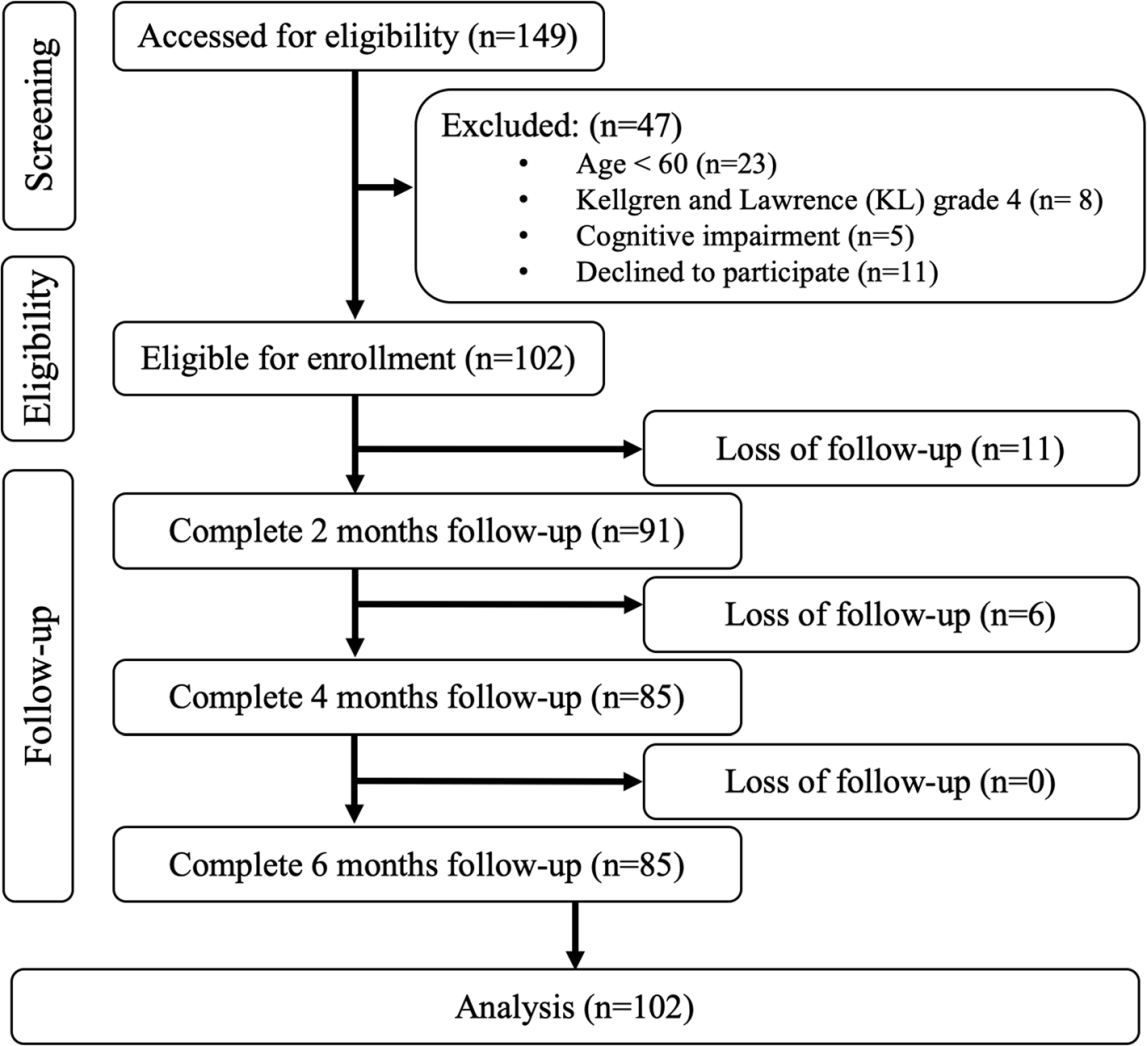
Patient eligibility chart

After screening 149 participants, 47 were excluded and the retained 102 participants all agreed to participate in this study (Fig. 1). Among the 102 participants, 74 (72.5%) were female, 80 received bilateral knee IAHA, and 22 undergone unilateral knee IAHA. Among the 182 knees within 102 patients, 20.3% of knees (*n* = 37) were defined as radiographic OA grade 3 on the Kellgren and Lawrence grading scale, whereas the other 145 knees were defined as radiographic OA grade 2. The mean age of the participants was 70.91 (SD = 7.19) years, and slightly more than one-quarter of them were males (*n* = 28). Almost all participants were married (*n* = 73), and nearly 30% of them (*n* = 30) had a bachelor’s or postgraduate degree. The most common comorbidity for the participants was hypertension (*n* = 63), which was followed by heart disease (*n* = 20), diabetes mellitus (*n* = 19), depression (*n* = 4), and cancer (*n* = 3). Table 1 provides the patient-reported outcomes and knee functions at four time points (baseline, and at 2-month, 4-month, and 6-month follow-ups).

**Table 1:** Descriptive statistics in patient-reported outcomes and knee functions across time (*N* = 102)

	*M* (SD)			
	Baseline	2 months	4 months	6 months
STAI^a^	20.22 (9.67)	19.14 (9.34)	19.47 (9.64)	19.07 (8.19)
GDS	2.75 (2.56)	2.99 (2.57)	2.96 (2.37)	2.87 (2.27)
VAS_L	4.46 (2.42)	3.02 (1.94)	3.81 (1.99)	4.23 (2.26)
VAS_R	4.23 (2.01)	3.04 (1.99)	4.04 (2.04)	4.42 (2.12)
IKDC	52.94 (13.23)	56.16 (12.28)	54.26 (12.17)	52.74 (12.12)
WOMAC pain	4.53 (3.99)	3.51 (3.28)	4.28 (3.37)	4.58 (3.64)
WOMAC stiffness	1.58 (1.70)	1.21 (1.45)	1.40 (1.47)	1.58 (1.51)
WOMAC physical function	14.75 (13.84)	11.78 (10.24)	12.98 (10.24)	13.95 (10.75)
WOMAC total score	20.85 (17.92)	16.52 (13.46)	18.67 (13.68)	20.09 (14.55)

*VAS_L*, Visual Analogue Scale on left knee pain; *VAS_R*, Visual Analogue Scale on right knee pain; *GDS*, Geriatric Depression Scale; *IKDC*, The International Knee Documentation Committee Questionnaire; *WOMAC*, Western Ontario and McMaster University Osteoarthritis Index

^a^State and trait anxiety inventory; only the state scale was used

When confounders were controlled for, age was significantly associated with IKDC score (coefficients [SEs] = − 0.317 [0.053]; *p* < 0.01), the WOMAC physical function subscale score (coefficients [SEs] = 0.393 [0.137]; *p* < 0.01), and the WOMAC physical function subscale score (coefficients [SEs] = 0.396 [0.174]; *p* < 0.05). Pain was significantly associated with the IKDC score (coefficients [SEs] = − 1.419 and − 1.337 [0.280 and 0.313]; *p* < 0.001, for left and right knee pain, respectively), the WOMAC pain subscale score (coefficients [SEs] = 0.280 and 0.362 [0.107 and 0.118]; *p* < 0.01, for left and right knee pain, respectively), the WOMAC stiffness subscale score (coefficient [SE] = 0.151 [0.053]; *p* < 0.01, for left right knee pain), the WOMAC physical function subscale score (coefficient [SE] = 1.158 [0.353]; *p* < 0.01, for right knee pain), and the WOMAC total score (coefficients [SEs] = 0.977 and 1.515 [0.421 and 0.470] for left and right knee pain; *p* < 0.05 and < 0.01, respectively). Anxiety was significantly associated with the WOMAC pain subscale score (coefficient [SE] = 0.056 [0.028]; *p* < 0.05). Depression was significantly correlated with the IKDC score (coefficient [SE] = − 1.064 [0.259]; *p* < 0.001), the WOMAC physical function subscale score (coefficient [SE] = 1.348 [0.292]; *p* < 0.001), and the WOMAC total score (coefficient [SE] = 1.595 [0.389]; *p* < 0.001) (Table 2).

**Table 2:** Effects of pain, anxiety, and depression on knee function

	*B* (SE)									
	IKDC		WOMAC_P		WOMAC_S		WOMAC_F		WOMAC_T	
VAS_L	− 1.419	(0.280)***	0.280	(0.107)**	0.151	(0.053)**	0.516	(0.316)	0.977	(0.421)*
VAS_R	− 1.337	(0.313)***	0.362	(0.118)**	0.055	(0.059)	1.158	(0.353)**	1.515	(0.470)**
STAI^a^	− 0.077	(0.072)	0.056	(0.028)*	0.003	(0.014)	0.093	(0.081)	0.148	(0.108)
Geriatric depression scale	− 1.064	(0.259)***	0.185	(0.098)^#^	0.054	(0.049)	1.348	(0.292)***	1.595	(0.389)***
2 months (Ref: baseline)	− 0.678	(0.867)	− 0.262	(0.347)	− 0.215	(0.175)	− 1.250	(0.971)	− 1.766	(1.306)
4 months (Ref: baseline)	− 0.314	(1.046)	− 0.007	(0.405)	− 0.129	(0.202)	− 1.015	(1.178)	− 1.171	(1.573)
6 months (Ref: baseline)	− 0.882	(1.156)	0.042	(0.433)	0.040	(0.215)	− 0.740	(1.311)	− 0.692	(1.736)
Gender (Ref: female)	3.878	(1.783)	0.631	(0.567)	− 0.397	(0.272)	− 1.617	(2.101)	− 1.417	(2.656)
Married (Ref: single)	− 14.214	(5.380)	1.513	(1.749)	0.914	(0.844)	2.258	(6.310)	4.633	(8.019)
Widowed (Ref: single)	− 17.211	(5.679)	2.404	(1.846)	0.818	(0.891)	3.957	(6.661)	7.133	(8.465)
Hypertension (Ref: yes)	1.085	(1.618)	− 0.322	(0.514)	0.188	(0.247)	− 0.990	(1.907)	− 1.184	(2.410)
DM (Ref: yes)	1.650	(1.954)	− 0.125	(0.617)	0.382	(0.296)	− 3.531	(2.305)	− 3.294	(2.908)
Heart disease (Ref: yes)	1.784	(2.007)	− 1.326	(0.640)	− 0.764	(0.308)	− 0.965	(2.363)	− 3.044	(2.989)
Depression (Ref: yes)	− 8.059	(6.055)	3.732	(1.961)	0.445	(0.946)	7.895	(7.107)	12.162	(9.024)
Cancer (Ref: yes)	3.241	(5.736)	− 2.932	(1.818)	0.536	(0.873)	− 5.338	(6.762)	− 7.969	(8.539)
Age	− 0.317	(0.117)**	− 0.023	(0.037)	0.025	(0.018)	0.393	(0.137)**	0.396	(0.174)*

*VAS_L*, Visual Analogue Scale on left knee pain; *VAS_R*, Visual Analogue Scale on right knee pain; *DM*, diabetes mellitus; *IKDC*, The International Knee Documentation Committee Questionnaire; *WOMAC*, Western Ontario and McMaster University Osteoarthritis Index

^#^*p* = 0.061; *p < 0.05; **p < 0.01; ***p < 0.001

^a^State and Trait Anxiety Inventory; only the state scale was used

As for the outcome comparisons between patients with Kellgren and Lawrence grades 2 and 3, patients with radiographic OA grade 3 were significantly associated with higher baseline GDS (*p* = 0.01) and lower baseline IKDC score (*p* = 0.01) than patients with radiographic OA grade 2 (Table 3). Additionally, during 6-month longitudinal follow-up, patients with radiographic OA grade 3 were also significantly associated with higher STAI, higher GDS, higher WOMAC score, and lower IKDC score than patients with radiographic OA grade 2 (Table 3).

**Table 3:** Outcome comparisons between patients with Kellgren and Lawrence grades 2 and 3

	Baseline			2 months			4 months			6 months		
	*M* (SD)		*t* (*p*)	*M* (SD)		*t* (*p*)	*M* (SD)		*t* (*p*)	*M* (SD)		*t* (*p*)
	Grade 2	Grade 3		Grade 2	Grade 3		Grade 2	Grade 3		Grade 2	Grade 3	
STAI^a^	18.94 (9.55)	23.29 (7.02)	1.93 (0.06)	16.92 (9.61)	25.05 (10.21)	**3.25 (0.002)**	17.78 (9.21)	23.95 (9.48)	**2.57 (0.01)**	17.49 (7.15)	22.55 (9.59)	**2.50 (0.02)**
Geriatric Depression Scale	2.36 (2.32)	3.86 (2.54)	**2.53 (0.01)**	2.41 (2.30)	4.25 (2.22)	**3.14 (0.002)**	2.41 (1.87)	4.30 (2.90)	**2.73 (0.01)**	2.34 (1.85)	4.15 (2.87)	**2.64 (0.01)**
VAS	4.23 (2.43)	5.14 (2.10)	1.55 (0.13)	2.70 (1.85)	3.90 (2.00)	**2.48 (0.02)**	3.59 (1.98)	4.20 (1.88)	1.20 (0.24)	4.08 (2.17)	4.80 (2.46)	1.23 (0.22)
IKDC	55.48 (13.58)	47.90 (9.26)	**2.91 (0.01)**	58.89 (11.81)	50.15 (11.08)	**2.92 (0.004)**	56.69 (11.84)	48.40 (11.30)	**2.74 (0.01)**	55.34 (11.55)	46.80 (12.36)	**2.81 (0.01)**
WOMAC pain	4.33 (4.09)	4.81 (3.53)	0.48 (0.63)	2.78 (2.55)	4.90 (4.12)	**2.18 (0.04)**	3.92 (3.11)	5.30 (4.13)	1.58 (0.12)	4.17 (3.56)	5.70 (4.17)	1.59 (0.12)
WOMAC stiffness	1.41 (1.73)	2.19 (1.44)	1.89 (0.06)	1.08 (1.42)	1.40 (1.31)	0.90 (0.37)	1.24 (1.47)	1.70 (1.38)	1.24 (0.22)	1.37 (1.50)	2.25 (1.41)	**2.30 (0.02)**
WOMAC physical function	13.17 (13.40)	19.33 (14.42)	1.81 (0.07)	9.24 (8.36)	17.15 (11.82)	**3.32 (0.001)**	11.31 (9.35)	17.80 (11.99)	**2.49 (0.02)**	12.46 (10.15)	17.90 (12.62)	1.95 (0.06)
WOMAC total score	18.91 (18.14)	26.33 (16.12)	1.68 (0.10)	13.13 (11.18)	23.45 (14.65)	**3.33 (0.001)**	16.47 (13.07)	24.80 (14.55)	**2.39 (0.02)**	17.98 (14.36)	25.85 (15.47)	**2.08 (0.04)**

*VAS*, Visual Analogue Scale; *DM*, diabetes mellitus; *IKDC*, The International Knee Documentation Committee Questionnaire; *WOMAC*, Western Ontario and McMaster University Osteoarthritis Index

Significant differences are shown in bold

^a^State and Trait Anxiety Inventory; only the state scale was used

## Discussion

This study demonstrates that knee pain and depression were the factors that most strongly and negatively affect the patient-reported knee functional outcomes, including IKDC and WOMAC scores, of elderly patients with knee OA who had undergone IAHA. Moreover, age is founded to be associated with poor patient-reported knee functional outcomes after IAHA. Additionally, patients with higher radiographic Kellgren and Lawrence grading (grade 3) presented with significantly more depression and poor knee functional outcomes than patients with lower radiographic OA grading (grade 2) at baseline and throughout 6-month longitudinal follow-up after IAHA.

Although IAHA is a commonly used intraarticular therapy for knee OA, controversy exists owing to the inconsistent results and conclusions of IAHA in the literature [33, 34]. Unanimity has not yet been reached on the usefulness of IAHA for treating knee OA [35]. A recently published network meta-analysis revealed that IAHA is an effective short-term treatment option for pain due to knee OA and even more efficacious than NSAIDs, intraarticular corticosteroid, or placebo injection [36]. However, the efficacy of IAHA among geriatric OA patients varies because of the inconsistent characteristics of this population. High prevalence of anxiety and depression has been reported among geriatric OA patients [9], but no studies listed anxiety and depression as independent psychological factors affecting the outcome after IAHA. In our study, although no effect of anxiety on self-reported knee functions was observed, the effects of depression were found to be one of independent factors affecting functional outcomes after IAHA. Although anxiety and depression are both psychological symptoms, depression is typically considered to be more severe than anxiety and is more like an affective disorder than a symptom [37]. Accordingly, depression, assessed using the GDS in this study, might have reflected the true mental health of the participants, whereas STAI (S-Anxiety) might have reflected subjective and transitory feelings of tension or the nervousness experienced by participants *at a given time*. Therefore, healthcare providers should pay more attention to depression rather than to anxiety for geriatric people with knee OA who have undergone IAHA.

In addition, pain was also found to be one of independent factors affecting functional outcomes after IAHA in our study. Along with depression, pain is one of the evaluated items in both IKDC and WOMAC. Therefore, the strong association between the patient-reported severity of pain based on VAS and the patient-reported function outcomes based on IKDC and WOMAC in this study is unsurprising. In addition, pain has been reported to be an independent factor to predict the worsening of OA [38]. Pre-operative knee pain sensitization or pain catastrophizing was also reported to be associated with patients’ satisfaction, quality of life, and functional improvement after total knee replacement [21, 39, 40]. Moreover, although only few studies discussed about the correction between pain and functional outcomes among geriatric OA patients receiving IAHA, Bowman EN et al. revealed that pain score was strongly correlated with successful outcomes and appeared to be a reliable method to monitor treatment success after IAHA [41]. Our study demonstrated the similar finding that knee pain was one of the factors negatively affecting the patient-reported knee functional outcomes after IAHA for elderly OA patients.

Moreover, age was also found to be one of the important factors affecting the efficacy of IAHA [7]. Evidence has demonstrated that elderly OA patients may not benefit from IAHA as much as younger patients, who experience a longer period of functional improvement of the knee [8, 42]. Hence, we postulated that the effects of IAHA are different between young-old OA patients (aged between 60 and 69 years) and old-old OA patients (aged 70 years or older). It is believed that elderly patients are vulnerable to worsen degeneration of cartilage as well as longer symptoms of knee OA so as to reflect the poor repose to IAHA. Our study also echoes the fact that age may negatively affect the patient-reported knee functional outcomes after IAHA. Additionally, more severe structural damage of knee was also an independent predictor for poor response to IAHA [8]. Indeed, our study demonstrated poor baseline and longitudinal self-reported functional outcome before and after IAHA in geriatric patients with higher radiographic OA grading. Also, these patients with more severe radiographic OA suffered more severe depressive status throughout the treatment duration with IAHA. Additional attention should therefore be paid to the potential influence of depression before IAHA especially among these geriatric OA patents with higher radiographic OA grading.

Our study has some limitations. First, the representativeness of our sample is somewhat questionable as we only recruited 102 elderly participants from the same institution, who might not represent the geriatric population with knee OA throughout Taiwan. Second, we did not exclude patients with other musculoskeletal disorders, except for knee OA. These musculoskeletal disorders might also contribute to chronic and subsequently affect the status of depression and anxiety among the geriatric population. Third, our causal relationship was not strong although a longitudinal design was adopted, and some degree of a causal relationship between psychological symptoms and knee functions was identified. Future studies using randomized controlled trials are warranted to corroborate our findings. Following the previous limitation, a longer period than our study follow-up is recommended because we believe that a 1-year follow-up could have been observed for more significant results. However, even with these limitations, this study is the first to show that psychological symptoms may contribute to poor knee function that is reported by geriatric patients with knee OA who are undergoing IAHA.

## Conclusion

Depression rather than anxiety was the most important psychological symptom that negatively affected the patient-reported knee functional outcomes of geriatric OA patients who had undergone IAHA. Further attention should be paid to the depressive status of geriatric people in cases of unsatisfactory outcomes of IAHA for knee OA that are reported by elderly patients.

## Data Availability

The datasets used and/or analyzed during the current study are available from the corresponding author on reasonable request.

## Abbreviations

GDS: Geriatric Depression Scale
IAHA: Intraarticular hyaluronic acid injection
IKDC: International Knee Documentation Committee subjective knee evaluation form
NSAIDs: Nonsteroidal anti-inflammatory drugs
OA: Osteoarthritis
SPMSQ: Short Portable Mental Status Questionnaire
STAI: State-Trait Anxiety Inventory
VAS: Visual Analogue Scale
WOMAC: Western Ontario and McMaster University Osteoarthritis Index
